# An evaluation of the preoperative hemoglobin level as a prognostic factor for oral squamous cell carcinoma

**DOI:** 10.1186/1758-3284-3-35

**Published:** 2011-08-15

**Authors:** Claudia Cordella, Heinz-Theo Luebbers, Valentina Rivelli, Klaus W Grätz, Astrid L Kruse

**Affiliations:** 1 Department of Craniomaxillofacial and Oral Surgery, University Hospital Zurich, (Frauenklinikstr. 24), Zürich, (CH-8091), Switzerland

## Abstract

**Background:**

Hypoxia seems to be an influencing factor for oral squamous cell carcinomas (SCC), and several immunohistochemical markers have been discussed in this regard. The aim of the present study was to evaluate preoperative hemoglobin levels as a prognostic factor for oral SCC.

**Materials and methods:**

The files of 287 patients who had been treated for oral SCC between 1999 and 2008 were studied retrospectively. Hemoglobin levels between 1 and 5 days prior to surgical treatment were compared to Tumor (T)- and Nodal (N)- status, local recurrence, and lymph node metastases rate. The minimum follow-up period was 12 months.

**Results:**

From a total of 287 patients with oral SCC, 205 (71.4%) were in the normal hemoglobin (Hb) group (female Hb≥12.0 g/dl; male Hb≥13.0 g/dl), 53 (18.5%) in the mild anemia (female Hb = 11.0-11.9 g/dl; male Hb = 11.0-12.9 g/dl), and 29 (10.1%) in the severe anemia group (female & male Hb<11.0 g/dl). Anemia was significant for the development of lymph node metastasis (p = 0.005) as well as for local recurrence (p = 0.001). No significant correlation was found to the initial T status (p = 0.183).

**Conclusion:**

Our data suggests that an Hb of below 11 g/dl contributes to and is an indicator for a poor prognosis. Consequently, pre-treatment Hb corrections may significantly improve outcome, but further investigations, including blood transfusion/application of erythropoietin due to tumor anemia, independent of intraoperative blood-loss are necessary to ascertain their role in an improved survival.

## Background

The estimated number of newly diagnosed cancers of the oral cavity and pharynx is 97,800 per year in Europe; the estimated number of deaths due to these carcinomas is 40,100 per year [1]. Despite improved diagnostic tools, chemotherapy, radiotherapy, and improved surgical techniques, the 5-year survival rate for head and neck cancer seems to be unchanged over the last two decades.

Clinical features and progression can differ greatly: some tumors develop no metastases, while others infiltrate at a very early stage and develop lymphangiomatosis or perineural invasion; such complications are not limited to well known risk factors such as smoking, alcohol consumption, poor mouth hygiene, or human papilloma virus (HPV). But the reason why the outcomes of some tumors are so much worse than those of others has still not been discovered. One of the most important prognostic factors for lymph node metastasis in head and neck cancer is tumor infiltration depth[2], but the reason for different histological growth patterns and lymphogenic metastases is still unknown.

Hypoxia seems to be an influencing factor for oral squamous cell carcinomas (SCC), and it is well known that the radiation doses needed to kill hypoxic cells are approximately 2-3 times higher than those needed to destroy well-oxygenated cells[3,4]. Most studies for head and neck SCC, however, deal with the effect of anemia as it relates to the outcome of radiotherapy[5,6]. Therefore, the question arises whether anemia also influences the outcome of surgical treatment. We have come across no studies which have dealt with this subject.

## Materials and methods

A total of 287 patients who were treated for oral SCC over a 10-year period between 1999 and 2008 at a single center (Clinic for Craniomaxillofacial and Oral Surgery, University Hospital Zurich) were evaluated retrospectively. All hemoglobin levels between 1 and 5 days prior to surgical treatment, Tumor (T)- and Nodal (N)-statuses, local recurrence rates, and lymph node metastases were taken into consideration. The minimum follow-up time was 12 months. Due to inadequate information and/or a follow-up time of less than 12 months, 82 patients were excluded.

Using the normal hemoglobin (Hb) values defined by the World Health Organization (WHO), the patients were divided into a three groups: normal (female Hb≥12.0 g/dl; male Hb≥13.0 g/dl), mild anemia (female Hb = 11.0-11.9 g/dl; male Hb = 11.0-12.9 g/dl), and severe anemia (female & male Hb<11.0 g/dl), according to Becker et al. [5]

All data were primarily obtained for medical purpose under informed consent of the patients. The study design fulfills the criteria of paragraphs 4a and b according to the guidelines (version 21.5.2010.2010) of the cantonal ethics committee of Zurich and therefore is exempted from institutional review board approval. The study design thereby fulfills the guidelines of the Declaration of Helsinki about Ethical Principles for Medical Research Involving Human Subjects.

For statistical analysis, SPPS 18 for Mac was used. P value < 0.05 was considered to be statistically significant. Kaplan-Meier analysis with log-rank testing was used for analysis.

## Results

**Figure 1:**
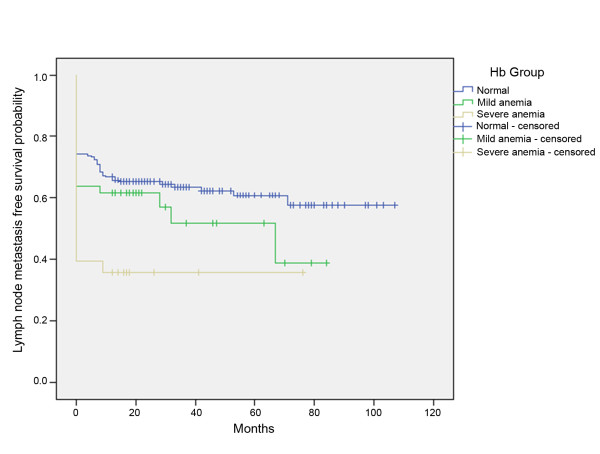
**Kaplan-Meier curves of lymph node metastasis -free survival for patients without anemia, mild anemia, or severe anemia**.

**Figure 2:**
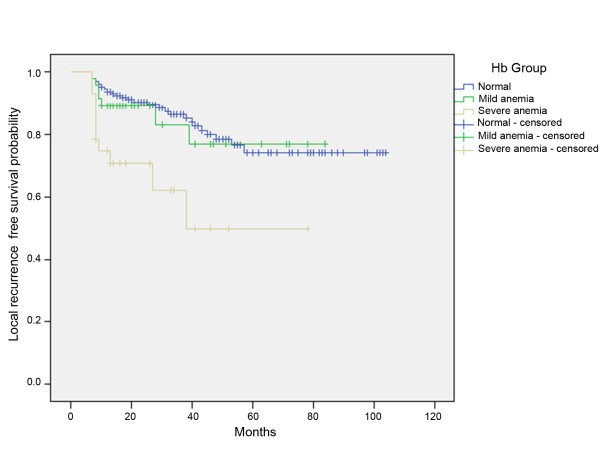
**Kaplan-Meier curves of local recurrence-free survival for patients without anemia, mild anemia, or severe anemia**.

Out of 287 patients with oral SCC, 205 (71.4%) were in the normal group, 53 (18.5%) in the mildly anemic, and 29 (10.1%) in the severely anemic group. The female-male ratio was 121:166, and the median age 62.53 years. In the log rank test, anemia was significant for the development of lymph node metastasis (p = 0.005) as well as for the development of local recurrence (p = 0.001) (Figure 1 and 2).

**Figure 3:**
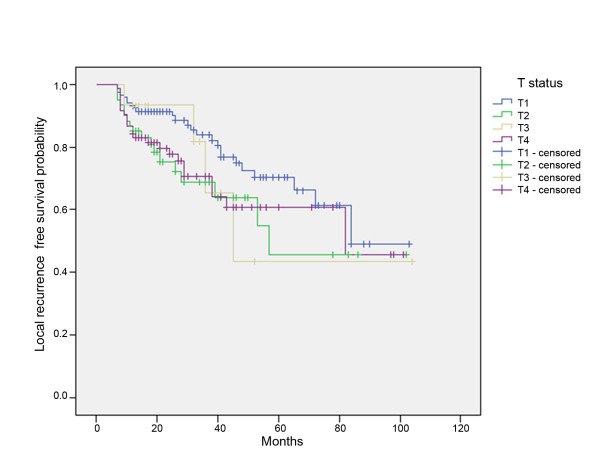
**Kaplan-Meier curves of local recurrence-free survival for patients with T1-, T2-, T3-, and T4-status**.

No association between T status and the development of local recurrence was found (p = 0.183) (Figure 3).

**Figure 4:**
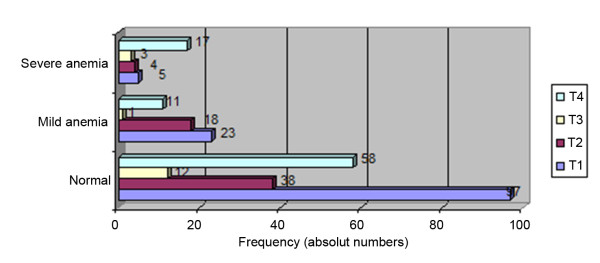
**Distribution of T status and grade of anemia**.

In the severe anemia group, 17 out of 29 patients (58.6%) had T4 status; in the normal group, 58 out of 205 patients (28.3%) had T4 status. There was a low number of patients with T3 status (Figure 4).

**Figure 5:**
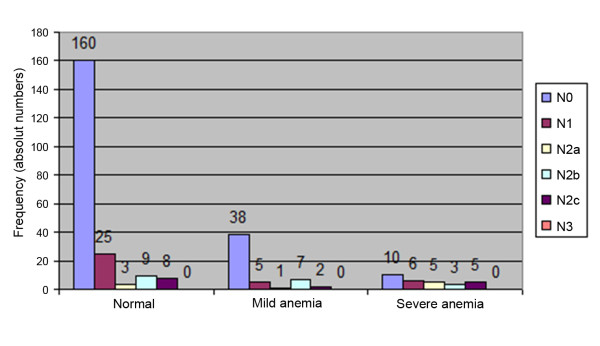
**Distribution of N status and grade of anemia**.

Lymph node metastases were observed in 45 out of 205 (28.1%) patients in the normal group and, in the severe anemia group, 19 out of 29 (65.5%) patients had positive lymph nodes (Figure 5).

## Discussion

It is widely accepted that anemia causes radiation resistance because the Hb level presumably mediates tumor response to radiation through the delivery of oxygen to the tumor[5]. There is increasing evidence that low Hb levels are indeed associated with poor tumor oxygenation, and increasing Hb concentrations are correlated with higher pO2 levels and lower hypoxic tissue fractions[7]. Therefore, several authors advocate blood transfusions before radiotherapy for anemic patients[8-10].

In the current study, low Hb levels were found to be associated with the development of lymph node metastasis (p = 0.005). Concerning the development of local recurrence, anemia is also a prognostic factor (p = 0.001), but, surprisingly, not the initial T status (p = 0.183). These data suggest that a low Hb concentration contributes to poor prognosis; these findings were supported by the results of several previous studies[11,12].

One reason could be that hypoxia is induced via Hypoxia-inducible Factor-1α (HIF-1α) and Vascular endothelial growth factor (VEGF), leading to a loss of p53[13].

If a low Hb concentration is a predictor of decreased local control, consequently, Hb corrections may significantly improve tumor oxygenation.

On the other hand, out of 29 patients in the severe anemia group, 17 had T4 tumors; therefore, large/infiltrative tumors might have led to anemia. However our data did not reveal T-status influence on local recurrence or lymph node metastases. Another reason could be the advanced stage of the disease. In the present study, 65.5% of patients with severe anemia had lymph node metastases, but, in the normal group, only 28.1% had positive lymph nodes.

However, if a lower Hb is associated with a worse outcome, one could conclude that blood transfusion could improve prognosis. To our knowledge, no study has dealt with this question. However, several studies have dealt with the need for intraoperative blood transfusion. (Table 1) This topic is not identical but somehow similar since low preoperative Hb-levels might - assumed surgical procedures are identical - lead to an intraoperative need for transfusion. However, evidence of a significant association between transfusion and prognosis in patients who undergo resection of oral SCC is conflicting: some authors have reported an influence [14,15], while others did not confirm blood transfusion as a prognostic factor[16,17]. After all, one must also differentiate between blood transfusions for preoperative anemia and those for surgical procedure/bleeding, as mentioned above.

**Table 1:** Studies regarding blood transfusion in head and neck cancer patients

Author	Patients (n)	Location	Outcome	Results
Böck[24]	174 (141 with transfusion)	Larynx/hypopharynx	Recurrence, survival, infection	No influence

Jones[25]	90 (46 with transfusion)	Head and neck	Recurrence	No influence

Ell[26]	240 (113 with transfusion)	Larynx	Survival	No influence

Woolley[27]	143 (99 with tranfusion)	Head and neck	Recurrence	P < 0.009

Von Doersten[28]	104 (51 with transfusion)	Head and neck	Recurrence, infection	No influence

Schuller[17]	217 (132 with transfusion)	Head and neck	Survival	No influence

Barra[29]	207 (152 with transfusion)	Head and neck	Survival	P < 0.05

Alun-Jones[30]	69 (38 with transfusion)	Larynx	Survival	P < 0.05

Sturgis[14]	61 (25 with transfusion)	Head and neck	Recurrence	Influence (48% with transfusion; 24% without transfusion)

Leon[31]	269 (86 with transfusion)	Larynx	Recurrence	No influence

Moir[32]	165 (60 with transfusion)	Head and neck	Recurrence	P < 0.04

Taniguchi[15]	105 (64 with transfusion)	Oral cavity	Survival	P < 0.01

Szakmany[33]	559 (437 with transfusion)	Oral cavity/ Oropharyngeal	Recurrence, survival	≥3 units influence

Fenner[16]	223	Oral cavity	Survival	No influence

Studies involving the effects of blood transfusion in head and neck cancer show inconsistent results

For patients with colorectal, lung, or breast carcinoma, an immunosuppressive effect of blood transfusions leading to increased local recurrence has been discussed, but is doubted by some authors[18,19], and it has not been proven so far for head and neck cancer. There is also some laboratory evidence for an association between blood transfusion and tumor growth[20].

One hypothesized mechanism for a worsened prognosis in tumor patients undergoing blood transfusion is the presence of biologically active growth factors in stored blood products. These factors seem to leach from red blood cells [21-23]

The strength of this study lays in the high number of patients with a minimum follow-up time (12 months) compared to smaller numbers of patients with head and neck cancer written about in the literature.

The limitation of the study is its retrospective nature. Conversely, the analysis was restricted to those variables that could be retrieved reliably for every patient from our database. Therefore, the number of blood transfusions and the amount of intraoperative blood-loss were excluded. In addition, other risk factors such as smoking, alcohol use, and poor mouth hygiene were not included because that information was not known. The number of patients with T3 status in this study is low and not consistent with T-status distribution of SCC found in many other studies and is not the distribution one would expect. However, this fact should not influence the results.

The idea of advocating preoperative transfusion or erythropoietin administration prior to surgery has very important economic as well as physiologic consequences and, given the grade of evidence, we consider this idea only with caution. Further investigations are needed, including examination of blood transfusion/application of erythropoietin due to tumor anemia in a prospective setting with multivariate analysis to rule out dependency with other more important factors. The influence of all negative side effects possibly coming with the transfusion of blood products also needs to be addressed[21].

Also there are several reasons why patients with head and neck tumors may have low preoperative hemoglobin, such as nutritional deficiency, difficulties involving oral intake, and/or social deprivation. Anemia could also be a marker for other risk factors, such as p53 mutation, loss of heterozygosity (LOH), HPV, etc. These factors should also be considered in future prospective studies.

In sum, the results of this study were not expected and no satisfying explanation is obvious. However, if it is found that Hb is not independent, it might still be an important and easily obtained marker for underlying conditions the cause for poor prognosis.

## Conclusion

Our data suggest that a low Hb level contributes to poor prognosis in oral SCC patients. Consequently, Hb corrections may significantly improve outcomes, but further investigations are necessary to clear the role of Hb in outcome prediction for oral SCC.

## Competing interests

The authors declare that they have no competing interests.

## Authors' contributions

VR and CC carried out the retrospective study, AK and CC drafted the manuscript, HTL drafted and finalized the manuscript, KG and AK participated in the design and coordination of the study.

All authors read and approved the final manuscript.
